# Performance d'un hôpital de zone sanitaire au Benin: un exemple de modèle d’évaluation

**DOI:** 10.11604/pamj.2014.18.63.3465

**Published:** 2014-05-18

**Authors:** Yolaine Glèlè Ahanhanzo, Landaogo Soutongonoma Lionel Ouédraogo, Jacques Saizonou

**Affiliations:** 1 Institut Régional de Santé Publique, Ouidah, Benin, BP 384 Ouidah, Bénin; 2 Direction régionale de la Santé du centre, Ministère de la Santé, Burkina Faso

**Keywords:** Evaluation, performance, fonctions, hôpital, Evaluation, performance, fonctions, hospital

## Abstract

**Introduction:**

Premier niveau de référence de la pyramide sanitaire du Bénin, les hôpitaux de zone sanitaire s'acquittent de leurs missions dans un contexte difficile. L'objectif de la présente étude a été d’évaluer la performance de l'hôpital de la zone sanitaire de Comè en 2013.

**Méthodes:**

L’étude était transversale, descriptive et évaluative. Les services retenus ont été sélectionnés par choix raisonné du fait de leur contribution au paquet d'activités de l'hôpital. Les clients externes et internes ont été sélectionnés par commodité. Les membres du conseil de gestion de l'hôpital de zone, les responsables d'organisation à base communautaire, les partenaires techniques et financiers ainsi que des chefs d'arrondissement ont été sélectionné par choix raisonné. La performance de l'hôpital a été mesurée à travers trois critères que sont la qualité des prestations, leur équité d'accès et leur pérennité. L'analyse des données a été faite sur la base de critères en utilisant une cotation analytique puis temporelle.

**Résultats:**

La performance de l'hôpital de la zone sanitaire de Comè était très faible au premier semestre 2013 avec une qualité des prestations cotée à 35%, une équité d'accès cotée à 50% et une pérennité des actions cotée à 11%. Seul le niveau d'application de la fonction gouvernance était moyen. La méconnaissance des attributions des représentants de la communauté dans les instances de l'hôpital a constitué une limite à leur implication dans l'exercice des fonctions de l'hôpital. Les partenaires techniques et financiers ont participé au renforcement institutionnel de l'hôpital en termes d'amélioration du plateau technique.

**Conclusion:**

L'application des fonctions de l'hôpital et une meilleure implication de la communauté ainsi que des partenaires contribueront à l'amélioration de la performance de l'hôpital de la zone sanitaire de Comè.

## Introduction

Depuis plus d'une décennie l'Organisation Mondiale de la Santé (OMS), la Banque Mondiale et d'autres organismes d'appui du secteur santé ont fait de l'amélioration des systèmes de santé une de leurs priorités. Les systèmes de santé se définissent comme toutes les ressources et activités dont le but essentiel est de promouvoir, restaurer ou entretenir la santé [1]. Répondre aux sollicitations de ses usagers, assurer une équité d'accès aux services et soins, et ainsi améliorer la santé des populations desservies constituent les objectifs de tout système de santé. Ces objectifs ne sont pas toujours atteints parce que cela dépend essentiellement des conditions dans lesquelles, ces systèmes mis en place parviennent à s'acquitter des six fonctions vitales que sont: la gouvernance, le financement, l'organisation et la gestion des ressources, les produits pharmaceutiques et consommables médicaux, les prestations de services et soins de santé, et le système d'information sanitaire [1–3]. Le potentiel des systèmes de santé n'est donc pas exploité et cela se traduit par un maintien de la morbidité et de la mortalité, des incapacités évitables, des inégalités et le non-respect des droits fondamentaux de la personne. Premier niveau de référence de la pyramide sanitaire du Bénin, l'hôpital en tant qu'unité du système de santé joue un rôle capital [4]. Le degré de réalisation de ses objectifs dépend essentiellement du niveau d'application de ses fonctions. La mise en application des fonctions de l'hôpital associé au degré d'implication de la communauté desservie et de ses partenaires techniques et financiers (PTF) dans l'exercice de ces fonctions vont contribuer à sa performance. Nous avons évalué le niveau d'application des fonctions de l'hôpital de zone de Comè (HZC) et le degré d'implication de la communauté et des PTF afin de déterminer et d'expliquer son niveau de performance en 2013.

## Méthodes

**Cadre d’étude:** L'hôpital de zone de Comè (HZC) est situé dans le sud est du Bénin. Il est la structure de première référence pour une population de 340.000 habitants. Il couvre une aire sanitaire de 1120 km^2^ couvrant 200 villages et disposant de 62 structures sanitaires de 1^er^ échelon parmi lesquelles 44 relèvent de l'autorité publique. Il est fonctionnel depuis une dizaine d'années, a une capacité d'accueil de 148 lits et offre le paquet complémentaire d'activités à travers les prestations de services et de soins curatifs, préventifs aussi bien généraux que spécialisés. L'HZC compte les services cliniques que sont les urgences, la gynécologie-obstétrique, la médecine interne, la pédiatrie, l'ophtalmologie, la stomato-odontologie et la kinésithérapie. Pour les services techniques ou d'appui, sont disponibles l'imagerie médicale, le laboratoire. Le secrétariat, la caisse, la pharmacie, les divisions maintenance, gestion malade et statistiques, hygiène et assainissement de base. Relèvent entre autres divisions de la direction administrative.

**Type d’étude:** Une étude transversale, descriptive et évaluative a été réalisée et a porté sur les activités de l'hôpital de zone de Comè du 1er semestre 2013.

**Population d’étude:** Elle était constituée par les cibles que sont les clients externes, les clients internes, les membres des organes de gestion de l'hôpital, les autorités locales, les représentants d'organisation à base communautaire, les partenaires techniques et financiers et des diverses sources telles que les services, les documents de planification, les outils de gestion et les infrastructures et équipements des services de l'HZC

**Echantillonnage:** Les services des urgences, de la maternité, de la chirurgie, du laboratoire et la direction ont été retenus par choix raisonné du fait de leurs contributions au paquet d'activités de l'hôpital de zone. Les clients externes et internes y ont été sélectionnés par commodité. Les infrastructures, les équipements biomédicaux et les outils de gestion des ressources ont été retenus de façon exhaustive. Les membres des organes de gestion de l'hôpital de zone, les représentants d'organisation à base communautaire, les partenaires techniques et financiers ainsi que les autorités locales ont été sélectionnés par choix raisonné

**Variables:** La variable principale de l’étude était la performance de l'hôpital de zone de Comè. Elle a été mesurée à travers les trois critères que sont la qualité des prestations, leur équité d'accès et leur pérennité. La qualité a été jugée sur les huit dimensions classiques: agrément, continuité, sécurité, efficacité, efficience, accessibilité, relations interpersonnelles, et compétences techniques. L’équité quant à elle, a été évaluée à travers l'existence de systèmes mutualistes, de systèmes de financement de soins aux populations indigentes et aux groupes cibles. La pérennité a été évaluée à travers la formation continue du personnel, le monitorage des activités, la capacité d'autofinancement, la part de la charge du personnel dans les dépenses, la mise en œuvre d'un plan de maintenance préventive pour les équipements, et l'effectivité d'un processus d'amélioration continue de la qualité. Les variables explicatives étaient le niveau d'application des six fonctions de l'hôpital: gouvernance, financement, organisation et gestion des ressources, produits pharmaceutiques, système d'information sanitaire, prestations de services et le degré d'implication de la communauté, des PTF dans la mise en œuvre des fonctions de l'hôpital. La fonction gouvernance a été évaluée sur la base du dispositif législatif et réglementaire, de la fonctionnalité des instances, de la disponibilité des normes et procédures et de la planification des activités. Les sources, les modes de financement et le bilan d'exécution budgétaire ont permis de juger la fonction financement tandis que l'organisation et la gestion des ressources a été évaluée par le respect des normes et standards, la gestion des ressources et la satisfaction générale des clients internes. La disponibilité et la tarification des médicaments et consommables médicaux, ainsi que la gestion des produits sanguins et dérivés ont permis d'apprécier la fonction produits pharmaceutiques. La fonction prestations des services et soins de santé a été évaluée à travers les quatre critères que sont la tarification des prestations de service, les activités de service et soins de santé, la prévention des infections et l'hôtellerie. Le système d'information sanitaire a été appréciée grâce à ses volets ressources, indicateurs, sources et gestion des données, produits d'information, diffusion et utilisation de l'information.

**Techniques et outils de collecte de données:** Les questionnaires, les guides d'entretien, les fiches de dépouillement et les grilles d'observation avec les techniques adaptées ont été utilisés pour la collecte.

**Traitement et analyse:** Les logiciels World, Excel et EPI info 2000 version 3.3.2 ont été utilisés pour l′analyse des données qui a été réalisée sur la bases de critères en utilisant une cotation analytique puis temporelle (Tableau 1). Pour obtenir le score global de performance, la qualité des soins et services a été pondérée à 50%, la pérennité et l’équité ont été pondérées chacune à 25%. La pondération des réalisations qui contribuent aux résultats d'ensemble de la performance a été faite à partir des variables explicatives. La cotation des critères d'appréciation a été effectuée sur la base des recommandations des documents normatifs nationaux [5] et du manuel d'accréditation des établissements de santé de la haute autorité de santé (HAS) de France [6].

**Tableau 1:** Modèle de cotation des critères de la performance et des fonctions de l'HZC au 1^er^ semestre 2013 suivant le modèle de cotation de la Haute Autorité de Santé de France

Analytique	La quasi-totalité des éléments d'appréciation du critère (≥80%)	La plupart des éléments d'appréciation du critère (60% –80%)	Quelques éléments d'appréciation du critère (40% - 60%)	Trop peu d’éléments d'appréciation du critère (< 40%)
	A	B	C	D
Régularité spatio-temporelle				
Quasi constant dans le temps (≥80%)	A	B	C	D
La plupart du temps (60 – 80%)	B	C	C	
Quelques fois (≤ 60%)	C	C	D	
Nulle part et/ou jamais	Pas de cotation			

Source: manuel de certification des établissements de santé et guide de cotation de la HAS [7].

Légende: A: niveau d'application de critère élevé, B: niveau d'application de critère moyen, C: niveau d'application de critère faible, D: niveau d'application de critère très faible.

Nous avons obtenu le consentement verbal de tous ceux qui ont été interrogées après information de la nature et des objectifs de l’étude.

## Résultats

**Figure 1:**
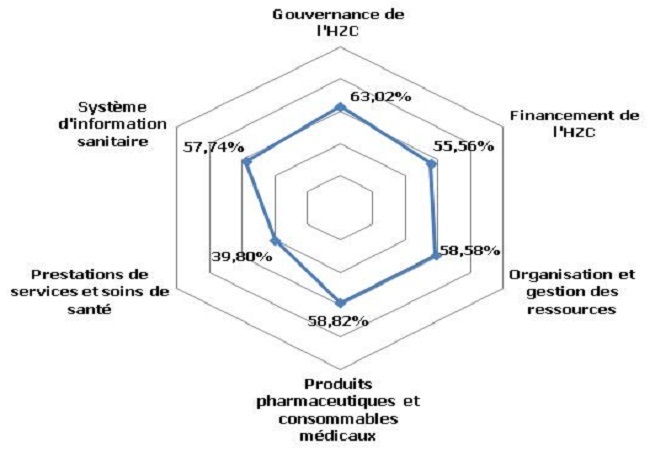
Degré de satisfaction des fonctions de l'hôpital de la zone sanitaire de Come au 1er semestre 2013

Pour l'analyse des six fonctions du système de santé de l'HZC, les résultats obtenus après la cotation analytique sont illustrés par la Figure 1. Après prise en compte de la cotation temporelle, la fonction de gouvernance cotée à 63.02% est finalement cotée C et donc jugée d'un niveau d'application faible de même que les fonctions financement, produits pharmaceutiques et système d'information sanitaire. Quant aux fonctions organisation et gestion des ressources et prestations des services initialement cotées respectivement à 58.6% et 39.9%, elles ont été jugées d'un niveau d'application très faible après analyse temporelle. Aucune fonction de l'HZC n'a atteint un niveau d'application élevé.

**Figure 2:**
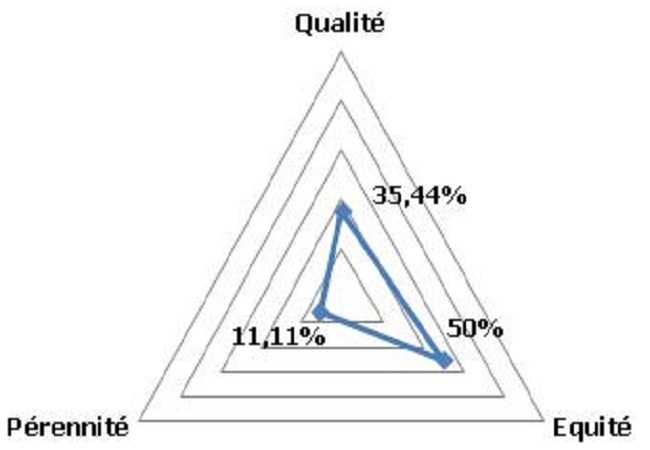
Degré de satisfaction des critères de la performance de l'HZC au 1er semestre 2013

Toutes les instances de l'HZC n’étaient pas mises en place, les actes administratifs n’étaient pas conformes aux textes en vigueur et seuls le conseil de gestion de l'hôpital et le comité de direction fonctionnaient. Le taux d'autofinancement était 23.26% et la mise en œuvre des directives d'exemption et de gratuité des prestations n’étaient pas permanente. Les différentes catégories professionnelles n’étaient pas aux normes et leur gestion souffrait de l'absence d'outils. De nombreuses ruptures des produits pharmaceutiques, des consommables médicaux, les produits sanguins et dérivés ont été enregistrées durant le 1^er^ semestre 2013. L'absence de contre références et d'activités de prévention vaccinale, ainsi que la non réalisation d'activités de recherche étaient les principales insuffisances du paquet complémentaire d'activités. Le développement du système d'information sanitaire était faible en particulier lié aux insuffisances de la production, diffusion et utilisation de l'information sanitaire. Les principaux indicateurs hospitaliers étaient: la proportion de femmes enceintes infectées par le VIH recevant une prophylaxie antirétrovirale complète pour réduire la transmission mère-enfant à 100%; le taux de césarienne à 4.7%; le taux d'infection du site opératoire après césarienne était de 2.41%; le taux de contre-référence était nul; le taux d'occupation des lits était de 105%; et le taux de satisfaction des clients externes à 46.12%. La prévention des infections et la gestion des déchets biomédicaux n’étaient pas satisfaisantes. Les représentants de la communauté dans le comité de gestion de l'hôpital ne connaissaient pas leurs attributions et n’étaient pas associés au processus de préparation des sessions statutaires. Les documents administratifs qui devaient porter la signature du président du CG/HZC n’étaient pas mis à jour. L'Hôpital de Zone de Comè avait plusieurs relations avec les autres acteurs de développement. Leurs domaines de partenariat étaient axés sur le renforcement institutionnel. Ces relations avec les PTF n’étaient pas toujours formalisées à l'aide d'un document type convention ou contrat de prestation. Les interventions de ces partenaires aussi bien techniques que financiers se faisaient suivant leurs domaines privilégiés ou à la demande de l'HZC. La démarche d'assurance qualité des soins n’était pas effective. Les directives d'exemptions et de gratuité ciblée des soins étaient appliquées. Il n'y avait pas d'alternative au paiement direct des prestations par les ménages. Il n'existait pas de plan de formation du personnel, ni de processus d'amélioration continue de la qualité des prestations à l'HZC. L'analyse de la performance de l'HZC a rapporté après la cotation analytique la qualité à 35.4%, l’équité à 50% et la pérennité à 11.1% (Figure 2). L'intégration de la cotation temporelle a permis de juger les niveaux de qualité et d’équité faible et le niveau de pérennité très faible. La performance de l'hôpital de la zone sanitaire de Comè a été jugée très faible au 1^er^ semestre 2013.

## Discussion

La présenté étude a porté sur l’évaluation de la performance de l'HZ de Comé. La performance des hôpitaux est un sujet d'actualité et de l'importance dans un contexte mondial marqué par la rareté des ressources. La notion de performance n'est pas unique. Notre modèle d’évaluation s'appuie sur trois concepts communs à la plupart des définitions proposés dans la littérature. Le concept de qualité des soins reste toutefois incontournable, même si être performant pour un établissement de santé signifie plus largement remplir ses missions en répondant à tous les besoins des usagers [7]. Selon la théorie des organisations, la performance hospitalière sera une résultante de l'application de quatre fonctions de l'organisation: i) s'adapter, puiser dans leurs environnements les ressources nécessaires pour offrir des services; ii) atteindre des buts qui sont valorisés (prévenir, diagnostiquer et traiter des problèmes de santé et des problèmes sociaux, réduire les inégalités à l’égard des soins et de la santé); iii) produire de façon intégrée, organiser la coordination entre les parties, assurer la qualité, être productif; iv) préserver et produire des valeurs et du sens [8]. La performance de l′hôpital doit être appréciée en fonction de la disponibilité des services pour tous les patients sans barrières physique, culturelle, sociale, démographique et économique » [9] Les notions d’équité et de pérennité justifient ainsi leur position dans notre modèle.

La gouvernance était moyenne eu égard à la non conformité des actes administratifs avec les textes législatifs et règlementaires, la non fonctionnalité de toutes les instances ainsi que l'absence de planification de l'AQS. Le niveau moyen de l'application de la fonction gouvernance entrave la notion de pouvoir partagé au sein de l'organisation qu'est l'hôpital de zone, afin de résoudre les problèmes de coordination des activités dans un environnement où évoluent des acteurs aux intérêts et logiques différentes selon Chauvancy [10]. Pour les alternatives au paiement direct des ménages, des opportunités existaient telles que la présence d'une multitude de mutuelles de santé dans la communauté desservie par l'HZC qui pourraient signer des conventions. Pour instaurer une certaine équité d'accès financier aux prestations de services et soins, des mécanismes d'exemption et de « gratuité » des soins étaient appliqués; néanmoins ces mécanismes sont ciblés pour les femmes enceintes ou les enfants de moins de cinq ans et sont bien entendu à l'origine d'une certaine inéquité par rapport aux autres cibles La disponibilité des ressources humaines dans notre étude ne respectait pas les normes nationales [11]. Nos résultats sont différents de ceux de Assavèdo [12] et de Tchindebet [13] qui avaient trouvés des effectifs de personnel répondant aux normes dans des services hospitaliers selon le document des normes et standards du ministère de la santé du Bénin qui date de 2001[14]. Les infrastructures et équipements disponibles correspondaient aux normes recommandées [14]. Le taux de satisfaction des clients internes était faible dû à la mauvaise appréciation des conditions de travail, de rémunération et de motivation. Les ruptures de produits pharmaceutiques et sanguins entravaient la mise en œuvre du paquet d'activités. Le niveau des indicateurs hospitaliers a révélé des insuffisances en rapport avec le non respect de la prévention des infections ainsi qu’à la gestion non adaptée des déchets biomédicaux. La faible performance du système d'information sanitaire de l'HZC entrave la planification et l'orientation objective des interventions. Le niveau d'implication de la communauté dans l'exercice des fonctions de l'hôpital était moyen et s'expliquerait par la méconnaissance de leurs attributions par les représentants et leur faible implication dans le processus de planification. L'HZC bénéficiait d'un réseau de partenaires multiples et variés qui l'accompagnaient dans ses missions. A titre d'exemple, des prestations étaient contractualisées avec des acteurs du développement local tels que les collectivités locales et les OBC. La matérialisation de ce partenariat n’était pas toujours faite par une convention de collaboration. Cependant ces acteurs se sentaient valorisés par ce partenariat qui leur permet d’être utiles à la communauté dans le domaine de la santé. Cela renforce le sentiment d'appartenance à sa communauté qui motive et améliore la performance de ces structures communautaires. Ce constat a été également fait par Malou [15] en 2012 sur l'effet bénéfique de l'image positive que la communauté a des relais communautaires qui participent à l'offre de soins dans la communauté. La performance de l'HZ de Comè était globalement très faible. La qualité des prestations était faible à cause principalement de l'absence de mise en œuvre du processus d'assurance qualité des soins. L’équité était faible et était liée essentiellement au défaut d'alternative de paiement direct des prestations bien que les mécanismes d'exemptions de gratuité des prestations étaient mis en œuvre selon les directives nationales. Le faible taux d'autofinancement et l'absence de processus d'amélioration continue de la qualité des prestations remettaient en cause la pérennité des interventions de l'hôpital. Suivant le guide national sur l’évaluation de la fonctionnalité et la performance des hôpitaux de zone [5], le score de performance de l'HZC était de 70,59% correspondant à l’échelle d'appréciation « assez-bien ». Ce score attribue une performance moyenne sur une échelle de cinq valeurs. La performance selon ces critères semble meilleure que celle trouvée dans notre étude. Cette variation s'explique par la différence entre les critères; l’évaluation nationale étant basée essentiellement sur des critères de disponibilité de ressources.

## Conclusion

La performance de l'HZC était très faible selon les critères de notre démarche d’évaluation axée sur le système de santé. Les insuffisances relevées au niveau de l'application des fonctions de l'hôpital peuvent être comblées par une planification rigoureuse associant l'ensemble des acteurs de l'HZC. La communauté devra être davantage informée sur les attributions de ses représentants au sein des organes de gestion de l'HZC. Les interventions des PTF devront être renforcées en ciblant les domaines prioritaires et ceux qui sont insuffisamment couverts par l'Etat afin de permettre à l'HZC de répondre au mieux à ses missions.
